# Prenatal and perinatal analgesic exposure and autism: an ecological link

**DOI:** 10.1186/1476-069X-12-41

**Published:** 2013-05-09

**Authors:** Ann Z Bauer, David Kriebel

**Affiliations:** 1 Department of Work Environment, School of Health and Environment, University of Massachusetts- Lowell, 1 University Avenue, Lowell, MA, 01854, USA

**Keywords:** Paracetamol, Acetaminophen, Autism spectrum disorder, Sulfation, Glucuronidation, Pro-inflammatory cytokines

## Abstract

**Background:**

Autism and Autism Spectrum Disorder (ASD) are complex neurodevelopmental disorders. Susceptibility is believed to be the interaction of genetic heritability and environmental factors. The synchronous rises in autism/ASD prevalence and paracetamol (acetaminophen) use, as well as biologic plausibility have led to the hypothesis that paracetamol exposure may increase autism/ASD risk.

**Methods:**

To explore the relationship of antenatal paracetamol exposure to ASD, population weighted average autism prevalence rates and paracetamol usage rates were compared. To explore the relationship of early neonatal paracetamol exposure to autism/ASD, population weighted average male autism prevalence rates for all available countries and U.S. states were compared to male circumcision rates – a procedure for which paracetamol has been widely prescribed since the mid-1990s. Prevalence studies were extracted from the U.S. Centers for Disease Control and Prevention Summary of Autism/ASD Prevalence Studies database. Maternal paracetamol usage and circumcision rates were identified by searches on Pub Med.

**Results:**

Using all available country-level data (n = 8) for the period 1984 to 2005, prenatal use of paracetamol was correlated with autism/ASD prevalence (r = 0.80). For studies including boys born after 1995, there was a strong correlation between country-level (n = 9) autism/ASD prevalence in males and a country’s circumcision rate (r = 0.98). A very similar pattern was seen among U.S. states and when comparing the 3 main racial/ethnic groups in the U.S. The country-level correlation between autism/ASD prevalence in males and paracetamol was considerably weaker before 1995 when the drug became widely used during circumcision.

**Conclusions:**

This ecological analysis identified country-level correlations between indicators of prenatal and perinatal paracetamol exposure and autism/ASD. State level correlation was also identified for the indicator of perinatal paracetamol exposure and autism/ASD. Like all ecological analyses, these data cannot provide strong evidence of causality. However, biologic plausibility is provided by a growing body of experimental and clinical evidence linking paracetamol metabolism to pathways shown to be important in autism and related developmental abnormalities. Taken together, these ecological findings and mechanistic evidence suggest the need for formal study of the role of paracetamol in autism.

## Background

Autism Spectrum Disorder (ASD) is a severe developmental disorder defined by social deficits, communication deficits, repetitive behaviors and fixated interests that appear in early childhood [1,2]. Despite a large and rapidly expanding body of literature, the etiology of ASD remains poorly understood. There is substantial evidence implicating oxidative stress, inflammation and immune dysregulation, although no single coherent explanation has emerged [3]. Recent twin studies provide evidence that susceptibility to ASD may have significant environmental components, in addition to genetic heritability [4,5].

Several lines of evidence suggest that medication use in pregnancy and early childhood may play a role in ASD etiology. Specifically, Torres and, Becker and Schultz, have hypothesized that paracetamol (acetaminophen, N-acetyl-p-aminophenol, or APAP) has increased ASD risk [6,7]. It has been shown that autistic children have a decreased capacity to sulfate paracetamol, which is the primary metabolic route for children [8]. When paracetamol is metabolized through the alternative routes it has been shown in humans to induce oxidative stress and immune dysregulation [9]. A recent investigation found transcriptomic changes in full-genome human miRNA expression indicating, for the first time, immune modulating effects and oxidative stress responses to paracetamol even at low doses [10]. Studies in animals have shown paracetamol to induce apoptosis and neurotoxicity [11,12]. Several studies hypothesize increased apoptosis in the autistic brain [13-16].

Paracetamol is one of the most common antipyretic analgesic medicines worldwide. In 1980, after sufficient evidence emerged of an association between salicylates and Reyes syndrome, paracetamol essentially replaced aspirin as the primary treatment of fever in children and pregnant women [17,18]. Since that date, paracetamol consumption throughout the world has increased dramatically [19]. Paracetamol sales in the United States (US) have had a continual upward trend. In 1982, US paracetamol sales were approximately $400 million; by 2008 they had risen to $2.6 billion [20,21]. Although prevalence data for autism and ASD are of uncertain accuracy, many authors report strong increases in prevalence over this same time period. Theoharides and colleagues for example reported prevalence prior to 1980 as approximately 4/10,000 and Baio et al. estimated that US autism/ASD prevalence to have risen to about 110/10,000 today [22,23].

Observing the correlation between two parallel time trends is of limited inferential utility; however the paracetamol – ASD link is strengthened by an observation first made by Becker and Schultz [7]. In 1982 and again in 1986, product tampering led to a few bottles of a leading brand of paracetamol tablets being contaminated with cyanide. In each case, a rapid and brief decline in paracetamol sales occurred, with the long term upward trend recovering within a year. In three populations for which good data are available, Becker and Schultz noted that brief dips in the rising autism prevalence curves mirrored these sales anomalies. The prevalence curves continued their upward trend after 1988.

### Study aims

Several lines of evidence suggest that the etiologically relevant period for the development of ASD may be *in utero* or possibly in early infancy [24,25]. We sought additional evidence to test the hypothesis that use of paracetamol in pregnancy or in early childhood might be a risk factor for ASD. Studies have reported population variation in prescribing patterns and usage rates which has allowed us an opportunity to investigate the correlation between prenatal exposure to paracetamol and autism spectrum disorder prevalence.

Additionally, we noted that analgesic prescribing habits for neonates and infants changed in the mid 1990’s with a shift in perspective on neonatal pain which has afforded us an additional opportunity to look at population variation in analgesic use [26]. Research beginning in the 1980’s documented the negative consequences associated with inadequate treatment of pain in children [27-29]. Pain guidelines specifically for children were established in 1992 by the Agency for Health Care Policy and Research, in 1998 by the World Health Organization and in 2001 by the American Academy of Pediatrics [30-33].

A common neonatal medical procedure is circumcision, which typically occurs during the postpartum hospital stay, within the first two days of life for a vaginal delivery and first four days for a cesarean section [34]. Prior to the 1990’s circumcision was generally performed without analgesics. A 1994 study by Howard et al. found that when paracetamol is given regularly every 6 hours for at least the first 24-hour postoperative period, infants demonstrated decreased responses to pain [35]. This study lead to the development of circumcision pain management guidelines by the American Academy of Pediatrics [36] and others [37-39]. These guidelines include the suggestion of a first dose of paracetamol two hours prior to the procedure, and doses every 4–6 hours for 24 hours following the procedure. Thus newborn males often receive 5–7 doses of paracetamol during the developmentally vulnerable initial days of life. Variations in circumcision frequency in different populations allowed us an additional approach to investigate the correlation between paracetamol use and ASD prevalence. This hypothesis seemed particularly relevant in light of the approximately 4.6 times higher prevalence of autism in males compared to females [23].

## Methods

Two separate analyses were conducted. The first examined the association between prenatal paracetamol exposure and ASD prevalence using maternal usage data as a proxy for prenatal exposure. The second analysis investigated the association between circumcision rates as a proxy for early life paracetamol exposure in males and ASD prevalence.

### Data for investigating prenatal paracetamol exposure and autism prevalence

To investigate the relationship of ASD to prenatal exposure to paracetamol, population maternal paracetamol usage rates were compared to population autism prevalence rates for as many countries as available data permitted. The population autism prevalence rates utilized were from studies reported in the U.S. Centers for Disease Control and Prevention Autism Prevalence Summary Table [40]. The maternal paracetamol usage rates by country were drawn from studies identified by a systematic search of the peer reviewed medical literature using the National Library of Medicine’s Pub Med database.

#### Autism prevalence rates

The Autism Prevalence Summary Table from the Center for Disease Control website [40] summarized the results of 59 prevalence studies conducted worldwide. This table recorded the author, year published, country, time period studied, age range studied, number of children in the population, the identification criteria, the methodology used and the prevalence. The oldest and youngest birth years in each study were calculated based on the time period and age range studied. Each study was extracted to verify the table results and to identify the ratio of males to females to calculate the male prevalence of autism/ASD. Generally, the CDC Prevalence Summary Table reported the more narrowly defined autism rate rather than the more inclusive diagnosis of autism spectrum disorder (ASD). The CDC Prevalence Summary reported the ASD rate for the Kim 2011 study in South Korea; however for consistency with all other results, the more narrowly defined autism rate was extracted and utilized in this analysis [41]. Two older studies referred to in the CDC table could not be located and were excluded (Lotter et al. 1966) (Brask et al. 1970). Two additional study were excluded, the first because of incomplete case ascertainment [42] and the second because of lack of total population data [43]. A total of 55 studies from the CDC summary were utilized in this analysis (Additional file 1).

#### Prenatal paracetamol usage rates

Maternal paracetamol usage rates were extracted from studies examining the use of paracetamol in pregnancy and relationships to other outcomes. A search of the English language literature on Pub Med was conducted for the past 20 years with a search date of April 18, 2012 using the terms prenatal, maternal, pregnancy, acetaminophen, paracetamol, medication, drugs, analgesic, pain relief, over the counter, and the different country names. Various combinations of the terms were used to maximize the results. If a study appeared to be relevant it was extracted and reviewed to identify a maternal paracetamol usage rate. If data were found only for overall analgesics, nervous system drugs, all over the counter medications or all western pharmaceuticals an assumption was made that paracetamol use represented 80%, 80%, 70% and 60%, respectively. These proportions were established a priori, conservatively approximated based on the findings of the US National Birth Defects Prevention Study and used for all studies regardless of country [18]. This literature search yielded 33 studies with medication usage rates for 14 out of 17 countries with autism prevalence rates. Two studies were excluded because they were subsets of two included studies to yield a total of 31 studies [44,45]. If multiple studies were identified for a country a summary usage rate was calculated using the weighted average by study population size. The characteristics of the prenatal medication usage studies are summarized in Additional file 2.

Because of concerns of changing autism prevalence rate over time, an a priori decision was made to restrict the analysis to include autism prevalence studies in which the range of birth years had at least one year of overlap with the range of birth years of the prenatal paracetamol usage studies. If multiple autism prevalence studies met this criterion for a given country, a weighted average based on study population size was calculated. This reduced the number of prenatal exposure studies used in this analysis to 20. There were inadequate data available to conduct a U.S. state level analysis.

### Data for investigating early life exposure to paracetamol for circumcision and autism prevalence

To investigate the relationship of early life paracetamol exposure for male neonates to autism spectrum disorder, population circumcision rates were compared to male population autism prevalence rates for two time periods. Male autism prevalence rates calculated from studies reported by the Center for Disease Control in the Summary of Autism/ASD Prevalence Studies (described above) were compared to male circumcision rates from studies identified by a systematic search of the peer reviewed medical literature using the National Library of Medicine’s Pub Med database. An additional U.S. state level analysis was done with available data (limited to the more recent time period) by comparing state and time period stratified male autism prevalence rates from the U.S. studies from the CDC Autism Prevalence Summary Table to newborn circumcision rates from the Health Care Utilization Project [46].

#### Circumcision rates

The circumcision rates were obtained by systematic search of the peer reviewed medical literature using the National Library of Medicine’s Pub Med database. A search of the English language literature on Pub Med was conducted using the terms circumcision and the different country names. The circumcision rates utilized the best identified information. If infant circumcision rates were available, they were utilized over national rates. If changing rates were presented, the rates for years closest to the study birth years were utilized. When a published paper was not available the rate was estimated. The estimation was calculated using the same methodology as the World Health Organization and the Circumcision Independent Reference and Commentary Service, calculated from the sum of the numbers of Jewish and Muslim males [47,48]. Data for the percentage of Jews by country were obtained from the Jewish Virtual Library [49]. Data for the percentage of Muslims by country were obtained from a Pew Forum report [50]. While most Jewish and Muslim males are circumcised, true circumcision rates are unknown and circumcision rates based upon religion are only an approximation. Both the World Health Organization and the Circumcision Independent Reference and Commentary Service indicated that this would likely underestimate the true prevalence (see Additional file 3). Annual U.S. state level infant circumcision rates were available for eight years between 1997 and 2006 from the Health Care Utilization Project (HCUP) of the Agency for Health Care Research and Quality (AHRQ) of the United States Department of Health and Human Services. Thirteen states had complete data for this eight year period and an additional seventeen states had partial data [46,51].

For the data analysis, the studies of autism prevalence were divided into two time periods. The first consisted of all prevalence studies in which all subjects were born before 1995 (35 country level studies). The assumption is that during this time period paracetamol would not generally have been administered during the circumcision procedure. The second, post-1995 cohort includes prevalence studies in which some subjects were born after 1994 (1995+), (20 country level studies) when circumcision pain was first recognized and treated. The assumption is that during this time period some portion of the cohort would have been administered paracetamol during the circumcision procedure. For countries with multiple studies in a time period, a summary prevalence was calculated using a weighted average based on study population size (Additional file 3). An additional U.S. state level analysis was conducted for the post-1995 cohort. Similarly, for states with multiple studies in a time period, a summary prevalence was calculated using a weighted average based on study population size. State level circumcision data were not available to conduct a pre-1995 analysis [Additional file 4]. Each of the data sets was checked for normality using standard graphical and statistical methods. Within the limits of these small datasets, the normality assumption was not seriously violated and so Pearson’s parametric correlation coefficient was used with an information weighted (1/variance) linear regression model.

## Results

### Prenatal exposure

**Figure 1:**
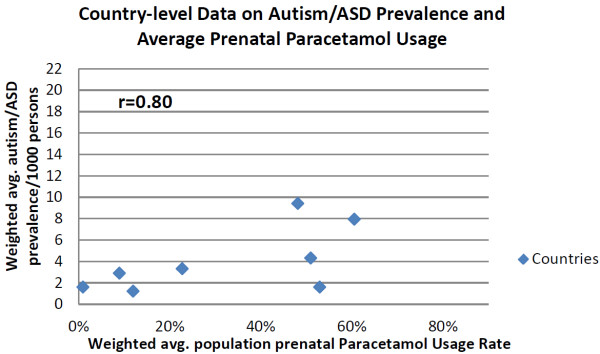
**Graph of country-level data on Autism/ASD prevalence and average prenatal paracetamol usage.** The Autism/ASD prevalence and the prenatal paracetamol usage rates are both population weighted averages of all the respective studies for a country. (See Additional files 2 and 5).

For the country-level analyses, synchronous data were available from 8 countries. A country’s average prenatal paracetamol consumption was found to be correlated with its autism/ASD prevalence (r = 0.80, Figure 1 and Additional file 5). The trend among the 8 countries indicates that a change of 10% in population prenatal paracetamol usage was associated with an increased autism population prevalence of 0.53/1000 persons (95% CI: 0.13 to 0.93) (Figure 1).

### Early life exposure

**Figure 2:**
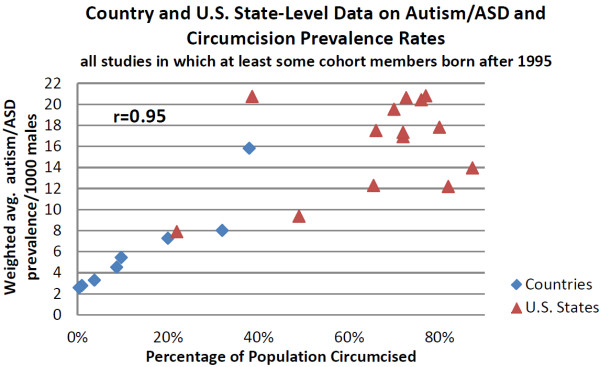
**Graph of country and U.S. state- level data on Autism/ASD and circumcision prevalence rates.** All studies in which at least some cohort members were born after 1995. This graph includes country-level studies with the U.S. stratified to state-level studies for the post-1995 cohort (no overall U.S. data point). The assumption is that, due to changes in neonate prescribing practices in the mid-1990s, some cohort members in each study would likely be exposed to paracetamol at the time of circumcision. Autism rates are population weighted averages of all studies for a country or U.S. state. (See Additional files 1, 3, 4 and 5).

A strong correlation (r = 0.98) was found in the country-level data between circumcision and autism spectrum disorder prevalence rates for boys born after 1995 (when circumcision guidelines began recommending analgesics). The slope of this trend for the 9 countries with available data indicates that a change of 10% in the population circumcision rate was associated with an increase in autism/ASD prevalence of 2.01/1000 persons (95% CI: 1.68 to 2.34) (Figure 2).

**Figure 3:**
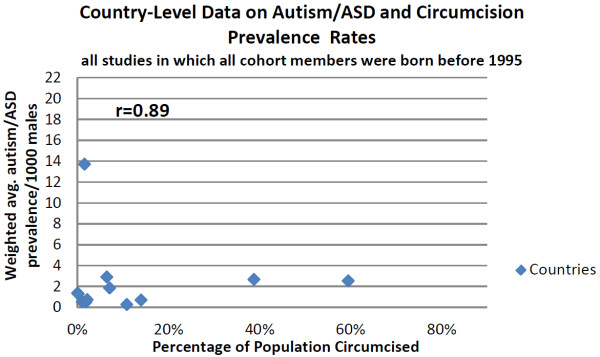
**Graph of country-level data on Autism/ASD and circumcision prevalence rates.** All studies in which all cohort members were born before 1995. The assumption is that all of the cohort members in each study would not likely be exposed to paracetamol at the time of circumcision. U.S. state level data was not available for this pre-1995 cohort. Autism rates are population weighted averages of all studies for a country. (See Additional files 1 and 3).

Data were available for 12 countries for boys born before 1995 (Figure 3), and the trend in the data was weaker; the correlation between circumcision prevalence and autism/ASD prevalence was still good (r = 0.89), but the slope of the trend was only a sixth of that for the later period for a 10% change in circumcision rate, there was an increase in autism/ASD prevalence of 0.35/1000 persons (95% CI: 0.22 to 0.47).

Across all country-level studies prior to the widespread use of paracetamol for circumcision (all born prior to 1995), the weighted average autism/ASD male to female prevalence ratio was 3.9 to 1. For the post 1995 cohort, this ratio increased to 5.6 to 1.

Available data allowed a parallel analysis of U.S. states post-1995, but not for the earlier period. The data for the 14 U.S. states with available data show a remarkably similar pattern to the 8 countries (this set of countries does not include the U.S. to avoid double counting) for the same time period (Figure 2, r = 0.95).

## Discussion

These ecological analyses identified positive correlations between autism/ASD prevalence and indicators of both prenatal and very early life paracetamol exposures. If these patterns are confirmed in formal epidemiologic studies, the use of paracetamol during pregnancy and at the time of circumcision may help to explain autism/ASD prevalence variations between the sexes, among countries, and within U.S. states and ethnic groups. The close to six-fold difference in rate of change in autism/ASD prevalence before and after the recognition of pain at the time of circumcision is suggestive of a possible effect caused by the shift to the use of paracetamol. The change in the overall time period average male to female autism/ASD prevalence ratio (weighted by study size) from 3.9 to 1 in the unexposed time period to 5.6 to 1 in the second time period with a probable paracetamol exposure may also be suggestive an effect from this exposure.

### Limitations

It is important to acknowledge that this analysis has numerous and significant limitations. First and foremost, correlation is not causation and as such no causal inference is intended. Homogeneity of exposure and prevalence assessment methodologies among the studies has been assumed, but each may be subject to misclassification, confounding and bias. The change in autism/ASD prevalence, circumcision prevalence and paracetamol usage rates over time may not have been adequately addressed. Circumcision rates are presented as a proxy for an early male neonatal exposure to paracetamol. However, this assumption is not without significant limitations. The timeline for the actual implementation of child pain management protocols and the utilization of paracetamol with circumcision is not known. Additionally, pain management guidelines suggest that paracetamol alone is not sufficient to manage circumcision pain so a nerve block or local anesthesia may also be administered, which may be confounding factors. In general, this type of ecologic study has significant limitations that severely limit causal inference. Ecologic bias or the failure of ecological associations to correspond to biologic effects at the individual level is a concern. It has been shown that the relations seen in country level data may poorly reflect the relationships that exist on an individual basis [52]. Despite these limitations, the consistent patterns reported here support the need to further investigate this potentially important hypothesis.

### Prenatal exposure trends

Previous research has identified paracetamol usage trends that curiously coincide with the rise in prevalence and population demographics of autism/ASD. In the US Slone Epidemiology Center Birth Defects study paracetamol was the most commonly used medication amongst all subjects with usage higher during pregnancy than before pregnancy. In the early 1980’s about 42% of women used paracetamol during the first trimester of pregnancy. The rate climbed to over 65% in the early 1990’s, where it has essentially remained through 2004 [18]. Maternal viral infection requiring hospitalization during the first trimester and maternal bacterial infection in the second trimester have been associated with diagnosis of ASD in the offspring (Hazard ratios 2.98 (95% CI: 1.29 to7.15) and 1.42 (95% CI: 1.08 to1.87), respectively) [53]. In a recent study, maternal self-reported influenza was associated with a twofold increased risk of infantile autism and a febrile episode lasting more than seven days was associated with a threefold increased risk [54]. Each of these maternal infections or a prolonged febrile episode would likely increase the exposure to paracetamol.

**Figure 4:**
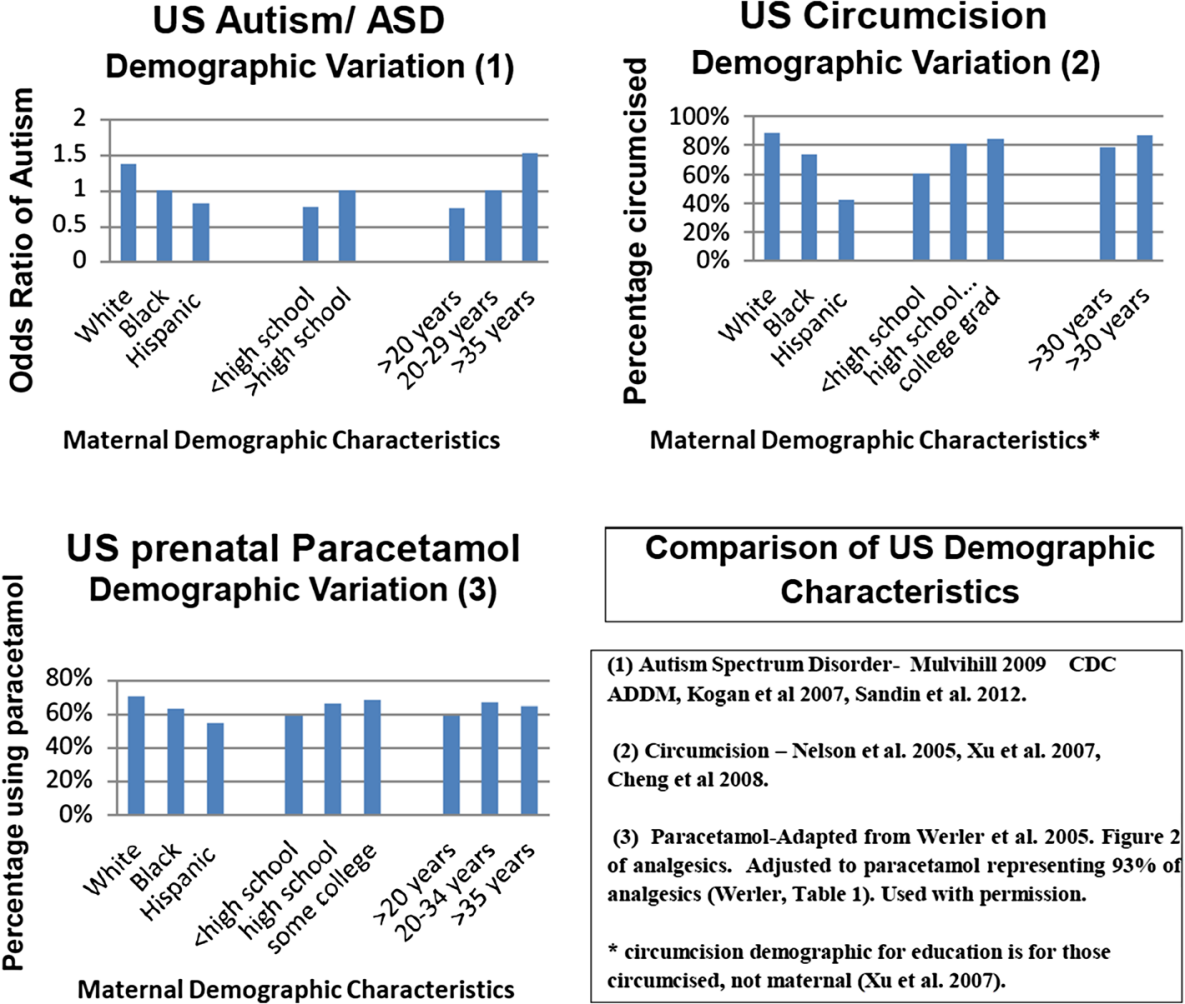
**Comparison of U.S. demographic characteristics.** Comparison of U.S. autism prevalence rates, circumcision rate and prenatal paracetamol usage rates by maternal race, education level and age.

In the U.S., usage of paracetamol by pregnant women mirrors the population demographics of women whose children develop autism spectrum disorder, by race, age and education [18,55,56]. The population demographics for mothers who circumcise their children are also very similar, with rates increasing with socio-economic status and insurance coverage rates [57-59]. Studies have shown that a parent’s own usage rates of paracetamol and other medications correlate with what they give to their children, so a similar demographic usage pattern would be expected for childhood exposure [60,61]. This synchronous U.S. pattern may be suggestive of an additive nature of both prenatal and early life exposure to paracetamol and a relationship to autism/ASD (Figure 4).

### Early life exposure trends

Paracetamol is the most common drug administered to US children and the predominant analgesic/antipyretic drug among children up to 24 months of age [62]. Paracetamol is suggested for pain management following vaccinations. In 1983 the average U.S. child received 8 immunizations before age 2. In 2011, the average was 25, a 313% increase [63,64]. From the perspective of the current hypothesis, these represent increased opportunities for paracetamol exposure in pain management (although administering several vaccines at once means analgesia may not increase proportionally).

A recent study representing one-fifth of all pediatric hospital admissions in the U.S., identified paracetamol as the most common drug administered to children over one year of age and the second most common drug administered for those under one year; more than 40% of hospital stays in both groups include paracetamol [65]. Hospitalization of children and neonates for infection as well as non-infectious disease have been associated with increased risk of ASD in a large Danish cohort (hazard ratios 1.38 (95% CI: 1.31 to 1.43) and 1.76 (95% CI: 1.68 to 1.86), respectively) [66].

### Biologic plausibility

**Figure 5:**
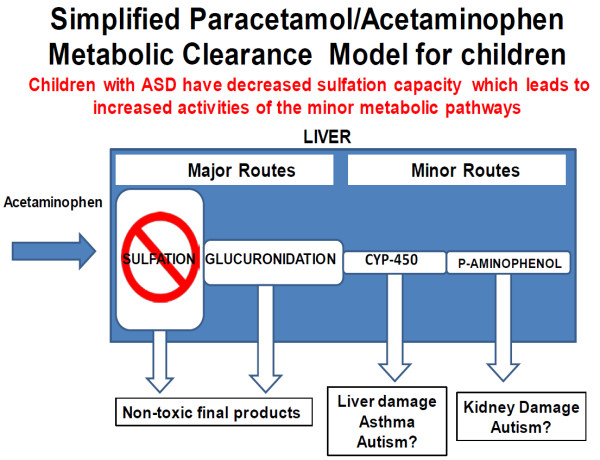
Metabolic pathway model for paracetamol.

Paracetamol has four important metabolic pathways (Figure 5). The two main pathways are glucuronidation and sulfation. Paracetamol is mainly metabolized in the liver via conjugation with glucuronide and sulfate and then excreted. Both these metabolic routes yield inactive, non-toxic final products. Glucuronidation is the primary metabolic pathway in adults and sulfation is the primary pathway for paracetamol metabolism until age 10–12 years [67]. Neonates, in general, have lower capacity to metabolize drugs due to the underdevelopment of the glucuronidation pathway and inefficiency and immaturity in renal function [68]. Three studies of neonates with postnatal age ranging from 1–3 days obtained a glucuronidation/sulfation (G/S) ratio between 0.12 and 0.28 [69-71]. This is in contrast to 11 month old children with a significantly higher G/S ratio of about 0.7 and adults with a G/S ratio approaching 2.0 [71,72]. Low birth weight and bilirubinemia have also been found to reduce glucuronidation capacity, both of which have been associated with autism [73-76]. Autistic children have been shown to have abnormal sulfate capacity and have been shown to have a specific inability to sulfate paracetamol [8,77,78]. Parents of autistic children have also been shown to have abnormal transsulfuration metabolism [79]. When the capacity to metabolize through the primary pathways is depleted or saturated, the fraction of the dose converted to reactive metabolites increases and the secondary metabolic pathways become increasingly involved [80].

One of the two secondary pathways is cytochrome P-450 (CYP P-450) mediated, forming a highly reactive metabolite, n-acetyl-p-benzoquinoneimine (NAPQI) which reacts with cellular glutathione (GSH) to form a non-toxic conjugate, which is subsequently excreted. Once GSH is exhausted, NAPQI binds to cellular proteins, including mitochondrial proteins reducing the ability to detoxify, which can lead to oxidative stress, immune system activation, hepatocellular death, nephropathy and asthma [10,19,81,82]. It has been shown that paracetamol treatment induces greater glutathione depletion in male mice [11,83]. Alterations in GSH homeostasis have been a consistent observation among autistic children and their mothers [84-87]. Also, decreased glutathione levels have been associated with preeclampsia [88]. In the Danish Birth Cohort, women who used paracetamol during the third trimester of pregnancy had increased risk of preeclampsia RR = 1.40 (95% CI: 1.24 to 1.58) [89]. In two studies, maternal preeclampsia has been associated with increased risk of having a child with ASD (OR = 1.69 (p = .0005) and RR = 1.64 (95% CI: 1.08 to 2.49), respectively) [90,91]. Additionally, during pregnancy a women’s sulfation capacity is reduced which may precipitate activation of immune responses, via this P-450 pathway [92,93]. The activation of immune response and pro- inflammatory cytokine interleukin signaling has recently been detected even at therapeutic doses of paracetamol [10,94]. Converging evidence highlights the important role of many of the same cytokines in mediating maternal immune activation effects on the neurodevelopment of autistic offspring [95-105].

A fourth metabolic pathway, accounting for about 6% of paracetamol metabolism, has been identified that is believed to be related to the mechanism of analgesic action [106]. This pathway involves deacetylation of paracetamol in the liver producing p-aminophenol that conjugates with arachidonic acid in the brain and in the spinal cord [107,108]. P-aminophenol is recognized to be involved in paracetamol nephrotoxicity [81,109]. More recently, P-aminophenol has been shown to produce a significant loss in mouse cortical neuron viability at therapeutic concentrations [12]. This suggests another possible pathway for a neurotoxic effect of paracetamol when the principle metabolic routes are exhausted.

In summary, there are several lines of evidence that suggest that prenatal and or early life paracetamol exposure may adversely affect neurodevelopment. Prenatal exposure may trigger maternal immune activation with possible effects on fetal brain development. In early life, maturational compromises to the glucuronidation pathway at the time of the circumcision related exposure, in combination with the compromises to the sulfation pathway that typify autistic children, may lead to utilization of the suboptimal secondary metabolic routes with the potential for adverse neurological effects in susceptible individuals [110-112].

## Conclusions

In this hypothesis generating exploratory analysis, several lines of evidence support the plausibility of a relationship between prenatal and early life exposure to paracetamol and autism spectrum disorder. It is proposed that the use of paracetamol in pregnancy and/or early childhood may alter immune processes increasing the risk of autism spectrum disorder in susceptible individuals. In an ecologic analysis, with all the previously discussed limitations, a correlation was found between maternal prenatal use of paracetamol and autism spectrum disorder. Additionally, a correlation was identified for the first time between neonatal circumcision with a probable paracetamol exposure and autism spectrum disorder. These relationships along with the synchronous rise in use of paracetamol and ASD, the convergence of the potential biologic mechanisms and the identification of plausible causes of increased male susceptibility provide consistent evidence of an association. Large scale population based epidemiologic studies are needed to confirm or disprove this association.

## Abbreviations

ASD: Autism Spectrum Disorder; CDC: Center for Disease Control, CIRCS.org, Circumcision Independent Reference and Commentary Service; CYP: Cytochrome; NAPQI: N-acetyl-p-benzoquinoneimine; GSH: Glutathione, AM404, N-arachidonoylphenolamine; Il: Interleukin; (G/S): Glucuronidation/sulfation.

## Competing interests

The authors declare that there are no conflicts of interest.

## Authors’ contributions

AB conceived of the study, made substantial contributions to its design and coordination, acquired the data, performed the data analysis and drafted the manuscript. DK made substantial contributions to concept and design, and revised the manuscript critically for intellectual content and data interpretation. Both authors read and approved the final manuscript.

## Supplementary Material

Additional file 1 **All Studies Reported in CDC Summary of Autism/ASD Prevalence Studies.** Summary of CDC studies divided into two groups. The first group has some members born post-1995 (some likely to be exposed to paracetamol at time of circumcision) and the second group born pre-1995 (exposure unlikely). The chart displays the study author, country, year published, range of birth years, ratio of males to females, study population, and the overall and male ASD prevalence [113-163]. Click here for file

Additional file 2 **Prenatal Paracetamol Exposure Studies Summary.** All prenatal paracetamol studies identified by a systematic search of the PubMed database. Studies without overlapping birth years with the autism prevalence studies are denoted with an “*” and were not utilized in the analysis. The chart displays the study author, country, year published, range of birth years, the study population, paracetamol usage rate, and study methodology [164-192]. Click here for file

Additional file 3 **Summary of Weighted average Autism studies and Circumcision Rates by Country.** Country weighted averages of CDC autism studies from Additional file 1. Data is divided into the two cohorts, pre and post −1995. The chart displays the circumcision rates and sources for each country and the study weights. The data in this table is used for the country-level analysis and comparison between the pre and post −1995 cohorts [193-206]. Click here for file

Additional file 4 **U.S. Studies Stratified by State from CDC Summary of Autism/ASD Prevalence Studies.** The six U.S. autism prevalence studies from Additional file 1 in the post 1995 cohort stratified by state. Chart displays the study author, country, year published, range of birth years, ratio of males to females, study population, the circumcision rates and sources, and the overall and male ASD prevalence. * Analysis restricted to 2006 data of 8 year olds. Click here for file

Additional file 5 **Summary of Weighted average Autism Prevalence and Prenatal Exposure to Paracetamol Data.** The Autism Prevalence Studies from Additional file 1 that had overlapping cohort birth years with the prenatal paracetamol studies in Additional file 2 are summarized. The study author, country, year published, range of birth years, population, overall ASD rate, the summary weighted average ASD rate by country, the weighted average APAP exposure rate,the APAP study range of birth years and the study weights. This data was used for Figure 1 and to calculate the prenatal correlation and trend. Click here for file

## References

[B1] Angley M Young R Ellis D Chan W McKinnon R Children and autism–Part 1– recognition and pharmacological management Aust Fam Physician 2007 36 741 744 17915375

[B2] Johnson CP Myers SM American academy of pediatrics council on children with disabilities. Identification and evaluation of children with autism spectrum disorders. Pediatrics 2007 120 1183 1215 10.1542/peds.2007-2361 17967920

[B3] Rossignol DA Frye REA Review of research trends in physiological abnormalities in autism spectrum disorders: immune dysregulation, inflammation, oxidative stress, mitochondrial dysfunction and environmental toxicant exposures Mol Psychiatry 2011 17 389 401 22143005 10.1038/mp.2011.165 PMC3317062

[B4] Ronald A Hoekstra RA Autism spectrum disorders and autistic traits: a decade of new twin studies Am J Med Genet B Neuropsychiatr Genet 2011 156B 255 274 21438136 10.1002/ajmg.b.31159

[B5] Hallmayer J Cleveland S Torres A Phillips J Cohen B Torigoe T Miller J Fedele A Collins J Smith K Lotspeich L Croen LA Ozonoff S Lajonchere C Grether JK Risch N Genetic heritability and shared environmental factors among twin pairs with autism Arch Gen Psychiatry 2011 68 1095 1102 10.1001/archgenpsychiatry.2011.76 21727249 PMC4440679

[B6] Torres AR Is fever suppression involved in the etiology of autism and neurodevelopmental disorders? BMC Pediatr 2003 3 9 10.1186/1471-2431-3-9 12952554 PMC194752

[B7] Becker KG Schultz ST Similarities in features of autism and asthma and a possible link to acetaminophen use Medical Hypotheses 2010 74 7 11 10.1016/j.mehy.2009.08.033 19748189 PMC3261751

[B8] Alberti A Pirrone P Elia M Waring RH Romano C Sulphation deficit in “low-functioning”autistic children: a pilot study Biol Psychiatry 1999 46 420 424 10.1016/S0006-3223(98)00337-0 10435209

[B9] Antoine DJ Jenkins RE Dear JW Williams DP McGill MR Sharpe MR Craig DG Simpson KJ Jaeschke H Park BK Molecular forms of HMGB1 and Keratin-18 as mechanistic biomarkers for mode of cell death and prognosis during clinical acetaminophen hepatotoxicity J Hepatol 2012 56 1070 1079 10.1016/j.jhep.2011.12.019 22266604 PMC4127883

[B10] Jetten MJ Gaj S Ruiz-Aracama A de Kok TM van Delft JH Lommen A van Someren EP Jennen DG Claessen SM Peijnenburg AA Stierum RH Kleinjans JC Omics analysis of low dose acetaminophen intake demonstrates novel response pathways in humans Toxicol Appl Pharmacol 2012 259 320 328 10.1016/j.taap.2012.01.009 22285215

[B11] Posadas I Santos P Blanco A Muñoz-Fernández M Ceña V Acetaminophen induces apoptosis in rat cortical neurons PLoS One 2010 5 15360 10.1371/journal.pone.0015360 PMC3000821 21170329

[B12] Schultz S Desilva M Gu TT Qiang M Whang K Effects of the analgesic acetaminophen (paracetamol) and its para-aminophenol metabolite on viability of mouse-cultured cortical neurons Basic & clinical pharmacology & toxicology 2011 Epub ahead of print 10.1111/j.1742-7843.2011.00767.x 21771276

[B13] Fatemi SH Earle JA McMenomy T Hippocampal CA4 Reelin-positive neurons Mol Psychiatry 2000 5 571 10.1038/sj.mp.4000794 11175329

[B14] Araghi-Niknam M Fatemi SH Levels of Bcl-2 and P53 are altered in superior frontal and cerebellar cortices of autistic subjects Cell Mol Neurobiol 2003 23 945 952 14964781 10.1023/B:CEMN.0000005322.27203.73 PMC11530152

[B15] Sheikh AM Li X Wen G Tauqeer Z Brown WT Malik M Cathepsin D and apoptosis related proteins are elevated in the brain of autistic subjects Neuroscience 2010 165 363 370 10.1016/j.neuroscience.2009.10.035 19854241

[B16] Malik M Sheikh AM Wen G Spivack W Brown WT Li X Expression of inflammatory cytokines, Bcl2 and cathepsin D are altered in lymphoblasts of autistic subjects Immunobiology 2011 216 80 85 10.1016/j.imbio.2010.03.001 20399529

[B17] Sullivan JE Farrar HC Fever and antipyretic use in children. Section on clinical pharmacology and therapeutics; committee on drugs Pediatrics 2011 127 580 587 10.1542/peds.2010-3852 21357332

[B18] Werler MM Mitchell AA Hernandez-Diaz S Honein MA Use of over-the-counter medications during pregnancy. The national birth defects prevention study Am J Obstet Gynecol 2005 193 771 777 10.1016/j.ajog.2005.02.100 16150273

[B19] Farquhar H Stewart A Mitchell E The role of paracetamol in the pathogenesis of asthma Clin Exp Allergy 2010 40 32 41 20205695 10.1111/j.1365-2222.2009.03378.x

[B20] Food and Drug Administration - Acetaminophen Background and Overview 2009 http://www.fda.gov/downloads/AdvisoryCommittees/CommitteesMeetingMaterials/Drugs/DrugSafetyandRiskManagementAdvisoryCommittee/UCM175767.pdf

[B21] Tylenol saleshttp://www.seegerweiss.com/drug-injury/tylenol/history-of-tylenol

[B22] Theoharides TC Kempuraj D Redwood L Autism an emerging ‘neuroimmune disorder’ in search of therapy Expert Opin Pharmacother 2009 10 2127 2143 10.1517/14656560903107789 19640207

[B23] Baio Wingate M Mulvihill B Kirby RS Pettygrove S Cunniff C Meaney F Schulz E Miller L Robinson C Quintana G Kaiser MY Lee LC Landa R Newschaffer C Constantino J Fitzgerald R Zahorodny W Daniels J Giarelli E Pinto-Martin J Levy SE Nicholas J Charles J Zimmerman J Maenner MJ Prevalence of autism spectrum disorders–autism and developmental disabilities monitoring network, 14 sites, united states, 2008. Autism and developmental disabilities monitoring network surveillance year 2008 principal investigators. Centers for disease control and prevention MMWR Surveill Summ 2012 61 1 19 22456193

[B24] Newschaffer CJ Fallin D Lee NL Heritable and nonheritable risk factors for autism spectrum disorders Epidemiol Rev 2002 24 137 153 10.1093/epirev/mxf010 12762089

[B25] Kolevzon A Gross R Reichenberg A Prenatal and perinatal risk factors for autism: a review and integration of findings Arch Pediatr Adolesc Med 2007 161 326 333 10.1001/archpedi.161.4.326 17404128

[B26] Schechter NL From the ouchless place to comfort central: the evolution of a concept Pediatrics 2008 122 S154 S160 10.1542/peds.2008-1055h 18978009

[B27] Mather L Mackie J The incidence of postoperative pain in children Pain 1983 15 271 282 10.1016/0304-3959(83)90062-3 6134266

[B28] Anand KJ Sippell WG Aynsley-Green A Randomised trial of fentanyl anaesthesia in preterm babies undergoing surgery: effects on the stress response Lancet 1987 1 62 66 2879174 10.1016/s0140-6736(87)91907-6

[B29] Fletcher AB Pain in the neonate N Engl J Med 1987 317 1347 1348 10.1056/NEJM198711193172110 3683464

[B30] AHCPR -Agency for Health Care Policy and Research Acute pain management in infants, children and adolescents: operative and medical procedures Am Fam Physician 1992 46 469 479 1636563

[B31] Anand KJ Consensus statement for the prevention and management of pain in the newborn Arch Pediatr Adolesc Med 2001 155 173 180 11177093 10.1001/archpedi.155.2.173

[B32] American Academy of Pediatrics: Committee on Psychosocial Aspects of Child and Family Health American pain society, task force on pain in infants children and adolescents. The assessment and management of acute pain in infants, children, and adolescents Pediatrics 2001 108 793 79711533354

[B33] WHO Normative Guidelines for Pain Management 2007 http://www.who.int/medicines/areas/quality_safety/delphi_study_pain_guidelines.pdf

[B34] Liu Z Dow WH Norton EC Effect of drive-through delivery laws on postpartum length of stay and hospital charges J Health Econ 2004 23 129 155 10.1016/j.jhealeco.2003.07.005 15154691

[B35] Howard CR Howard FM Weitzman ML Acetaminophen analgesia in neonatal circumcision: the effect on pain Pediatrics 1994 93 641 646 8134222

[B36] American Academy of Pediatrics Circumcision policy statement task force on circumcision Pediatrics 1999 103 686 693 10049981

[B37] Geyer J Ellsbury D Kleiber C Litwiller D Hinton A Yankowitz J An evidence-based multidisciplinary protocol for neonatal circumcision pain management JOGNN 2002 31 403 410 10.1111/j.1552-6909.2002.tb00062.x 12146929

[B38] Macke JK Analgesia for circumcision: effects on newborn behavior and mother/infant interaction JOGNN 2001 30 507 514 10.1111/j.1552-6909.2001.tb01570.x 11572531

[B39] Melnyk BM Fineout-Overholt E Evidence-based practice in nursing & healthcare 2005 Philadelphia, PA: Lippincott, Williams, & Wilkins

[B40] Center for Disease Control Autism Prevalence Studies http://www.cdc.gov/ncbddd/autism/documents/Autism_PrevalenceSummaryTable_2011.pdf

[B41] Kim YS Leventhal BL Koh YJ Fombonne E Laska E Lim EC Cheon KA Kim SJ Kim YK Lee H Song DH Grinker RR Prevalence of autism spectrum disorders in a total population sample Am J Psychiatry 2011 168 904 912 10.1176/appi.ajp.2011.10101532 21558103

[B42] Al-Farsi YM Al-Sharbati MM Al-Farsi OA Al-Shafaee MS Brooks DR Waly MI Brief report: prevalence of autistic spectrum disorders in the sultanate of Oman J Autism Dev Disord 2011 41 821 825 10.1007/s10803-010-1094-8 20809376

[B43] Gurney JG Fritz MS Ness KK Sievers P Newschaffer CJ Shapiro EG Analysis of prevalence trends of autism spectrum disorder in Minnesota Arch Pediatr Adolesc Med 2003 157 622 627 10.1001/archpedi.157.7.622 12860781

[B44] Feldkamp ML Meyer RE Krikov S Botto LD Acetaminophen use in pregnancy and risk of birth defects: findings from the national birth defects prevention study Obstet Gynecol 2010 115 109 115 10.1097/AOG.0b013e3181c52616 20027042

[B45] Gunawardana L Zammit S Lewis G Gunnell D Hollis C Wolke D Harrison G Examining the association between maternal analgesic use during pregnancy and risk of psychotic symptoms during adolescence Schizophr Res 2011 126 220 225 10.1016/j.schres.2010.10.021 21146371

[B46] Healthcare Cost and Utilization Project (HCUP) Agency for Health Care Policy and Research (US) http://www.hcup-us.ahrq.gov/databases.jsp 21413206

[B47] Circumcision Independent Reference and Commentary Service http://www.circs.org/index.php/Reviews/Rates/Global

[B48] WHO Library: Male circumcision: global trends and determinants of prevalence, safety and acceptabilityhttp://whqlibdoc.who.int/publications/2007/9789241596169_eng.pdf

[B49] Jewish Virtual Library. The Jewish Population of the World http://www.jewishvirtuallibrary.org/jsource/Judaism/jewpop.html

[B50] Pew Forum. Mapping the Global Muslim Population http://pewforum.org/newassets/images/reports/Muslimpopulation/Muslimpopulation.pdf

[B51] Incidence of Circumcision by State http://www.cirp.org/library/statistics/USA/state-by-state/

[B52] Rothman KJ Greenland S Lash T Seigafuse S Modern Epidemiology 2008 3 Philadelphia: Lippincott Williams and Wilkins 511 531

[B53] Atladottir H Thorsen P Ostergaard L Schendel D Lemcke S Abdallah M Parner E Maternal infection requiring hospitalization during pregnancy and autism J Autism Dev Disord 2010 40 1423 1430 10.1007/s10803-010-1006-y 20414802

[B54] Atladóttir HÓ Henriksen TB Schendel DE Parner ET Autism after infection, febrile episodes, and antibiotic use during pregnancy: an exploratory study Pediatrics 2012 2012 e1447 e1454 Spectrum Disorder. *J Autism Dev Disord* 2010, 40:1423–1430 23147969 10.1542/peds.2012-1107 PMC4451062

[B55] Kogan MD Blumberg SJ Schieve LA Boyle CA Perrin JM Ghandour RM Singh GK Strickland BB Trevathan E van Dyck PC Prevalence of parent-reported diagnosis of autism spectrum disorder among children in the US, 2007 Pediatrics 2009 124 1395 1403 10.1542/peds.2009-1522 19805460

[B56] Sandin S Hultman CM Kolevzon A Gross R Maccabe JH Reichenberg A Advancing maternal age is associated with increasing risk for autism: a review and meta-analysis J Am Acad Child Adolesc Psychiatry 2012 51 477 486 10.1016/j.jaac.2012.02.018 22525954

[B57] Nelson CP Dunn R Wan J Wei JT The increasing incidence of newborn circumcision: data from the nationwide inpatient sample J Urol 2005 173 978 981 10.1097/01.ju.0000145758.80937.7d 15711354

[B58] Cheng D Hurt L Horon IL Neonatal circumcision in Maryland: a comparison of hospital discharge and maternal postpartum survey data J Pediatr Urol 2008 4 448 451 10.1016/j.jpurol.2008.06.007 18691938

[B59] Xu F Markowitz LE Sternberg MR Aral SO Prevalence of circumcision and herpes simplex virus type 2 infection in men in the United States: the National Health and Nutrition Examination Survey (NHANES), 1999–2004 Sex Transm Dis 2007 34 479 484 17413536 10.1097/01.olq.0000253335.41841.04

[B60] Rasmussen F Smedby B Physician visits and prescribed drugs among young children and their mothers Scand J Prim Health Care 1987 5 225 231 10.3109/02813438709018100 3423493

[B61] Jensen JF Tønnesen LL Söderström M Thorsen H Siersma V Paracetamol for feverish children: parental motives and experiences Scand J Prim Health Care 2010 28 115 120 10.3109/02813432.2010.487346 20470019 PMC3442316

[B62] Vernacchio L Kelly JP Kaufman DW Mitchell AA Medication use among children <12 years of age in the United States: results from the Slone Survey Pediatrics 2009 124 446 454 10.1542/peds.2008-2869 19651573

[B63] Center for Disease Control 1983 vaccine schedulehttp://www.cdc.gov/vaccines/schedules/images/schedule1983s.jpg 1983 vaccine schedule

[B64] Center for Disease Control 2011 vaccine schedule http://www.cdc.gov/vaccines/recs/acip 2011 vaccine schedule

[B65] Feudtner C Dai D Hexem KR Luan X Metjian TA Prevalence of polypharmacy exposure among hospitalized children in the United States Arch Pediatr Adolesc Med 2012 166 9 16 10.1001/archpediatrics.2011.161 21893637

[B66] Atladóttir HO Thorsen P Schendel DE Østergaard L Lemcke S Parner ET Association of hospitalization for infection in childhood with diagnosis of autism spectrum disorders: a Danish cohort study Arch Pediatr Adolesc Med 2010 164 470 477 10.1001/archpediatrics.2010.9 20439799

[B67] Schultz ST Can autism be triggered by acetaminophen activation of the endocannabinoid system? Acta Neurobiol Exp (Wars) 2010 70 227 231 20628445 10.55782/ane-2010-1793

[B68] Allegaert K de Hoon J Verbesselt R Vanhole C Devlieger H Tibboel D Intra- and interindividual variability of glucuronidation of paracetamol during repeated administration of propacetamol in neonates Acta Paediatr 2005 94 1273 1279 16278989 10.1111/j.1651-2227.2005.tb02088.x

[B69] Van Lingen RA Deinum JT Quak JM Kuizenga AJ van Dam JG van Dam JG Anand KJ Tibboel D Okken A Pharmacokinetics and metabolism of rectally administered paracetamol in preterm neonates Arch Dis Child Fetal Neonatal Ed 1999 80 F59 F63 10.1136/fn.80.1.F59 10325815 PMC1720876

[B70] Levy G Khanna NN Soda DM Tsuzuki O Stern L Pharmacokinetics of acetaminophen in the human neonate: formation of acetaminophen glucuronide and sulfate in relation to plasma bilirubin concentration and D-glucaric acid excretion Pediatrics 1975 55 818 825 1134883

[B71] Miller RP Roberts RJ Fischer LJ Acetaminophen elimination kinetics in neonates, children and adults Clin Pharmacol Ther 1976 19 284 294 1261167 10.1002/cpt1976193284

[B72] Van der Marel CD Anderson BJ Van Lingen RA Holford NH Pluim MA Jansman FG van den Anker JN Tibboel D Paracetamol and metabolite pharmacokinetics in infants Eur J Clin Pharmacol 2003 59 243 251 10.1007/s00228-003-0608-0 12761605

[B73] Allegaert K van den Anker J Pharmacokinetics and pharmacodynamics of intravenous acetaminophen in neonates Expert Rev Clin Pharmacol 2011 4 713 718 10.1586/ecp.11.50 22111857

[B74] Allegaert K Palmer GM Anderson BJ The pharmacokinetics of intravenous paracetamol in neonates: size matters most Arch Dis Child 2011 96 575 580 10.1136/adc.2010.204552 21317433

[B75] Gardener H Spiegelman D Buka SL Perinatal and neonatal risk factors for autism: a comprehensive meta-analysis Pediatrics 2011 128 344 355 10.1542/peds.2010-1036 21746727 PMC3387855

[B76] Amin SB Smith T Wang H Is neonatal jaundice associated with autism spectrum disorders: a systematic review J Autism Dev Disord 2011 41 1455 1463 10.1007/s10803-010-1169-6 22009628 PMC4285414

[B77] Kern JK Ramsden DB Grannemann BD Garver CR Ryaskin OT Abnormal Sulfation Chemistry in Autism Trends in Autism Research 2004 New York: Nova Biomedical Books 211

[B78] Yap IK Angley M Veselkov KA Holmes E Lindon JC Nicholson JK Urinary metabolic phenotyping differentiates children with autism from their unaffected siblings and age-matched controls J Proteome Res 2010 9 2996 3004 10.1021/pr901188e 20337404

[B79] James SJ Melnyk S Jernigan S Hubanks A Rose S Gaylor DW Abnormal transmethylation/transsulfuration metabolism and DNA hypomethylation among parents of children with autism J Autism Dev Disord 2008 38 1966 1975 10.1007/s10803-008-0591-5 18512136 PMC2584168

[B80] Zhao L Pickering G APAP metabolism and genetic differences Drug Metab Rev 2011 43 41 52 10.3109/03602532.2010.527984 21108564

[B81] Louis J Curtis D John B Klaassen CD Toxic Responses of the Liver Casarett and Doull’s Essentials of Toxicology- The Basic Science of Poisons. 7th 2008 New York: McGraw-Hill 569 571

[B82] Dimova S Hoet PHM Dinsdale D Newery B Acetaminophen decreases intercellular glutathione levels and modulate cytokine production in human alveolar macrophages and type II pneumocytes in vitro Int J Bio Chem Cell Biol 2004 37 1727 1737 10.1016/j.biocel.2005.03.005 15878691

[B83] McConnachie LA Mohar I Hudson FN Ware CB Ladiges WC Fernandez C Chatterton-Kirchmeier S White CC Pierce RH Kavanagh TJ Glutamate cysteine ligase modifier subunit deficiency and gender as determinants of acetaminophen-induced hepatotoxicity in mice Toxicol Sci 2007 99 628 636 10.1093/toxsci/kfm165 17584759

[B84] Williams TA Mars AE Buyske SG Stenroos ES Wang R Wang R Factura-Santiago MF Lambert GH Johnson WG Risk of autistic disorder in affected offspring of mothers with a glutathione S-transferase P1 haplotype Arch Pediatr Adolesc Med 2007 161 356 361 17404132 10.1001/archpedi.161.4.356

[B85] Ballatori N Krance SM Notenboom S Shi S Tieu K Hammond CL Glutathione dysregulation and the etiology and progression of human diseases Biol Chem 2009 390 191 214 19166318 10.1515/BC.2009.033 PMC2756154

[B86] Bowers K Li Q Bressler J Avramopoulos D Newschaffer C Fallin MD Glutathione pathway gene variation and risk of autism spectrum disorders J Neurodev Disord 2011 3 132 143 10.1007/s11689-011-9077-4 21484198 PMC3188290

[B87] Maher P Methylglyoxal, advanced glycation end products and autism: is there a connection? Med Hypotheses 2012 78 548 552 10.1016/j.mehy.2012.01.032 22325990

[B88] Oken E Ning Y Rifas-Shiman SL Rich-Edwards JW Olsen SF Gillman MW Diet during pregnancy and risk of preeclampsia or gestational hypertension Ann Epidemiol 2007 17 663 668 10.1016/j.annepidem.2007.03.003 17521921 PMC2532559

[B89] Rebordosa C Zelop CM Kogevinas M Sørensen HT Olsen J Use of acetaminophen during pregnancy and risk of preeclampsia, hypertensive and vascular disorders: a birth cohort study J Matern Fetal Neonatal Med 2010 23 371 378 10.3109/14767050903334877 19929241

[B90] Mann JR McDermott S Bao H Hardin J Gregg A Pre-eclampsia, birth weight, and autism spectrum disorders J Autism Dev Disord 2010 40 548 554 10.1007/s10803-009-0903-4 19936906

[B91] Buchmayer S Johansson S Johansson A Hultman CM Sparén P Cnattingius S Can association between preterm birth and autism be explained by maternal or neonatal morbidity? Pediatrics 2009 124 e817 e825 10.1542/peds.2008-3582 19841112

[B92] Davies MH Ngong JM Yucesoy M Acharya SK Mills CO Weaver JB Waring RH Elias E The adverse influence of pregnancy upon sulphation: a clue to the pathogenesis of intrahepatic cholestasis of pregnancy? J Hepatol 1994 21 1127 1134 10.1016/S0168-8278(05)80630-0 7699239

[B93] Lee JK Abe K Bridges AS Patel NJ Raub TJ Pollack GM Brouwer KL Sex-dependent disposition of acetaminophen sulfate and glucuronide in the in situ perfused mouse liver Drug Metab Dispos 2009 37 1916 1921 10.1124/dmd.109.026815 19487254 PMC2729328

[B94] Wright G Shawcross D Olde Damink SW Jalan R Brain cytokine flux in acute liver failure and its relationship with intracranial hypertension Metab Brain Dis 2007 22 375 388 10.1007/s11011-007-9071-4 17899343

[B95] Deverman BE Patterson PH Cytokines and CNS development Neuron 2009 64 61 78 Review 10.1016/j.neuron.2009.09.002 19840550

[B96] Abdallah MW Larsen N Grove J Nørgaard-Pedersen B Thorsen P Mortensen EL Hougaard DM Amniotic fluid inflammatory cytokines: potential markers of immunologic dysfunction in autism spectrum disorders World J Biol Psychiatry 2011 Epub ahead of print 10.3109/15622975.2011.639803 22175527

[B97] Goines PE Croen LA Braunschweig D Yoshida CK Grether J Hansen R Kharrazi M Ashwood P Van de Water J Increased midgestational IFN-gamma, IL-4 and IL-5 in women bearing a child with autism: a case–control study Mol Autism 2011 2 13 10.1186/2040-2392-2-13 21810230 PMC3170586

[B98] Jyonouchi H Sun S Le H Proinflammatory and regulatory cytokine production associated with innate and adaptive immune responses in children with autism spectrum disorders and developmental regression J Neuroimmunol 2001 120 170 179 10.1016/S0165-5728(01)00421-0 11694332

[B99] Vargas DL Nascimbene C Krishnan C Zimmerman AW Pardo CA Neuroglial activation and neuroinflammation in the brain of patients with autism Ann Neurol 2005 57 67 81 Erratum in: *Ann Neurol* 2005, 57:304 10.1002/ana.20315 15546155

[B100] Li X Chauhan A Sheikh AM Patil S Chauhan V Li XM Ji L Brown T Malik M Elevated immune response in the brain of autistic patients J Neuroimmunol 2009 207 111 116 10.1016/j.jneuroim.2008.12.002 19157572 PMC2770268

[B101] Cai Z Pan ZL Pang Y Evans OB Rhodes PG Cytokine induction in fetal rat brains and brain injury in neonatal rats after maternal lipopolysaccharide administration Pediatr Res 2000 47 64 72 10.1203/00006450-200001000-00013 10625084

[B102] Bell MJ Hallenbeck JM Effects of intrauterine inflammation on developing rat brain J Neurosci Res 2002 70 570 579 10.1002/jnr.10423 12404511

[B103] Shi L Fatemi SH Sidwell RW Patterson PH Maternal influenza infection causes marked behavioral and pharmacological changes in the offspring J Neurosci 2003 23 297 302 12514227 10.1523/JNEUROSCI.23-01-00297.2003 PMC6742135

[B104] Patterson PH Immune involvement in schizophrenia and autism: etiology, pathology and animal models Behav Brain Res 2009 204 313 321 10.1016/j.bbr.2008.12.016 19136031

[B105] Shi L Tu N Patterson PH Maternal influenza infection is likely to alter fetal brain development indirectly: the virus is not detected in the fetus Int J Dev Neurosci 2005 23 299 305 10.1016/j.ijdevneu.2004.05.005 15749254

[B106] Högestätt ED Jönsson BA Ermund A Andersson DA Björk H Alexander JP Cravatt BF Basbaum AI Zygmunt PM Conversion of acetaminophen to the bioactive N-acylphenolamine AM404 via fatty acid amide hydrolase-dependent arachidonic acid conjugation in the nervous system J Biol Chem 2005 280 31405 31412 10.1074/jbc.M501489200 15987694

[B107] Mallet C Daulhac L Bonnefont J Ledent C Etienne M Chapuy E Libert F Eschalier A Endocannabinoid and serotonergic systems are needed for acetaminophen-induced analgesia Pain 2008 139 190 200 10.1016/j.pain.2008.03.030 18485596

[B108] Bertolini A Ferrari A Ottani A Guerzoni S Tacchi R Leone S Paracetamol: new vistas of an old drug CNS Drug Rev 2006 12 250 275 10.1111/j.1527-3458.2006.00250.x 17227290 PMC6506194

[B109] Gemborys MW Mudge GH Formation and disposition of the minor metabolites of acetaminophen in the hamster Drug Metab Dispos 1981 9 340 351 6114834

[B110] Van den Anker JN Pharmacokinetics and renal function in preterm infants Acta Paediatr 1996 85 1393 1399 10.1111/j.1651-2227.1996.tb13942.x 9001646

[B111] McCarver DG Hines RN The ontogeny of human drug metabolizing enzymes: phase II conjugation enzymes and regulatory mechanisms J Pharmacol Exp Ther 2002 300 361 366 10.1124/jpet.300.2.361 11805192

[B112] Kearns GL Abdel-Rahman SM Alander SW Blowey DL Leeder JS Kauffman RE Developmental pharmacology–drug disposition, action, and therapy in infants and children N Engl J Med 2003 349 1157 1167 10.1056/NEJMra035092 13679531

[B113] Parner ET Thorsen P Dixon G de Klerk N Leonard H Nassar N Bourke J Bower C Glasson EJ A comparison of autism prevalence trends in Denmark and Western Australia J Autism Dev Disord 2011 41 1601 1608 10.1007/s10803-011-1186-0 21311963

[B114] Williams E Thomas K Sidebotham H Emond A Prevalence and characteristics of autistic spectrum disorders in the ALSPAC cohort Dev Med Child Neurol 2008 50 672 677 10.1111/j.1469-8749.2008.03042.x 18754916

[B115] Icasiano F Hewson P Machet P Cooper C Marshall A Childhood autism spectrum disorder in the Barwon region: a community based study J Paediatr Child Health 2004 40 696 701 10.1111/j.1440-1754.2004.00513.x 15569287

[B116] Ouellette-Kuntz H Coo H Lloyd JE Kasmara L Holden JJ Trends in special education code assignment for autism: implications for prevalence estimates J Autism Dev Disord 2007 37 1941 1948 10.1007/s10803-006-0326-4 17216561

[B117] Lauritsen MB Pedersen CB Mortensen PB The incidence and prevalence of pervasive developmental disorders: a Danish population-based study Psychol Med 2004 34 1339 1346 10.1017/S0033291704002387 15697060

[B118] Chakrabarti S Fombonne E Pervasive developmental disorders in preschool children JAMA 2001 285 3093 3099 10.1001/jama.285.24.3093 11427137

[B119] Baron-Cohen S Scott FJ Allison C Williams J Bolton P Matthews FE Brayne C Prevalence of autism-spectrum conditions: UK school-based population study Br J Psychiatry 2009 194 500 509 Erratum in: Br J Psychiatry: 195, 182 10.1192/bjp.bp.108.059345 19478287

[B120] Lingam R Simmons A Andrews N Miller E Stowe J Taylor B Prevalence of autism and parentally reported triggers in a north east London population Arch Dis Child 2003 88 666 670 10.1136/adc.88.8.666 12876158 PMC1719604

[B121] Baird G Charman T Baron-Cohen S Cox A Swettenham J Wheelwright S Drew A A screening instrument for autism at 18 months of age: a 6-year follow-up study J Am Acad Child Adolesc Psychiatry 2000 39 694 702 10.1097/00004583-200006000-00007 10846303

[B122] Wong VC Hui SL Epidemiological study of autism spectrum disorder in China J Child Neurol 2008 23 67 72 18160559 10.1177/0883073807308702

[B123] Chien IC Lin CH Chou YJ Chou P Prevalence and incidence of autism spectrum disorders among national health insurance enrollees in Taiwan from 1996 to 2005 J Child Neurol 2011 26 830 834 10.1177/0883073810393964 21460178

[B124] Rice C Nicholas J Baio J Pettygrove S Lee LC Van Naarden Braun K Doernberg N Cunniff C Newschaffer C Meaney FJ Charles J Washington A King L Kolotos M Mancilla K Mervis CA Carpenter L Yeargin-Allsopp M Changes in autism spectrum disorder prevalence in 4 areas of the United States Disabil Health J 2010 3 186 201 10.1016/j.dhjo.2009.10.008 21122784

[B125] Pinborough-Zimmerman J Bakian AV Fombonne E Bilder D Taylor J McMahon WM Changes in the administrative prevalence of autism spectrum disorders: contribution of special education and health from 2002–2008 J Autism Dev Disord 2011 42 521 530 21538173 10.1007/s10803-011-1265-2

[B126] Windham GC Anderson MC Croen LA Smith KS Collins J Grether JK Birth prevalence of autism spectrum disorders in the San Francisco Bay area by demographic and ascertainment source characteristics J Autism Dev Disord 2011 41 1362 1372 10.1007/s10803-010-1160-2 21264681

[B127] Bertrand J Mars A Boyle C Bove F Yeargin-Allsopp M Decoufle P Prevalence of autism in a United States population: the Brick Township, New Jersey, investigation Pediatrics 2001 108 1155 1161 10.1542/peds.108.5.1155 11694696

[B128] Mulvihill B Wingate M Kirby RS Pettygrove S Cunniff C Meaney FJ Miller L Robinson C Quintana G Kaiser MY Lee LC Landa R Newschaffer C Constantino J Fitzgerald R Daniels J Giarelli E Pinto-Martin J Levy SE Charles J Nicholas J Durkin M Rice C Baio J Van Naarden BK Yeargin-Allsopp M Hepburn M Garner N Mancilla KC Ratchford A CDC ADDM -Autism and Developmental Disabilities Monitoring Network Surveillance Year 2006 Principal Investigators; Centers for Disease Control and Prevention (CDC). Prevalence of autism spectrum disorders - Autism and Developmental Disabilities Monitoring Network, United States, 200658:1–20 MMWR Surveill Summ 2009 58 1 20 Erratum in: *MMWR Surveill Summ* 59:956 20023608

[B129] Montiel-Nava C Peña JA Epidemiological findings of pervasive developmental disorders in a Venezuelan study Autism 2008 12 191 202 10.1177/1362361307086663 18308767

[B130] Bryson SE Clark BS Smith IM First report of a Canadian epidemiological study of autistic syndromes J Child Psychol Psychiatry 1988 29 433 445 10.1111/j.1469-7610.1988.tb00735.x 3265136

[B131] Fombonne E Zakarian R Bennett A Meng L McLean-Heywood D Pervasive developmental disorders in Montreal, Quebec, Canada: prevalence and links with immunizations Pediatrics 2006 118 e139 150 10.1542/peds.2005-2993 16818529

[B132] Kočovská E Biskupstø R Carina Gillberg I Ellefsen A Kampmann H Stórá T Billstedt E Gillberg C The rising prevalence of autism: a prospective longitudinal study in the faroe islands J Autism Dev Disord 2012 42 1959 66 10.1007/s10803-012-1444-9 22271195

[B133] Wing L Gould J Severe impairments of social interaction and associated abnormalities in children: epidemiology and classification J Autism Dev Disord 1979 9 11 29 10.1007/BF01531288 155684

[B134] Baird G Simonoff E Pickles A Chandler S Loucas T Meldrum D Charman T Prevalence of disorders of the autism spectrum in a population cohort of children in South Thames: the Special Needs and Autism Project (SNAP) Lancet 2006 368 210 215 10.1016/S0140-6736(06)69041-7 16844490

[B135] Fombonne E Is there an epidemic of autism? Pediatrics 2001 107 411 412 10.1542/peds.107.2.411 11158478

[B136] Powell JE Edwards A Edwards M Pandit BS Sungum-Paliwal SR Whitehouse W Changes in the incidence of childhood autism and other autistic spectrum disorders in preschool children from two areas of the West Midlands, UK Dev Med Child Neurol 2000 42 624 628 10.1017/S001216220000116X 11034456

[B137] Webb EV Lobo S Hervas A Scourfield J Fraser WI The changing prevalence of autistic disorder in a Welsh health district Dev Med Child Neurol 1997 39 150 152 9112962 10.1111/j.1469-8749.1997.tb07402.x

[B138] Kielinen M Linna SL Moilanen I Autism in Northern Finland Eur Child Adolesc Psychiatry 2000 9 162 167 10.1007/s007870070039 11095038

[B139] Cialdella P Mamelle N An epidemiological study of infantile autism in a French department (Rhône): a research note J Child Psychol Psychiatry 1989 30 165 175 10.1111/j.1469-7610.1989.tb00775.x 2784446

[B140] Fombonne E Du Mazaubrun C Cans C Grandjean H Autism and associated medical disorders in a French epidemiological survey J Am Acad Child Adolesc Psychiatry 1997 36 1561 1569 9394941 10.1016/S0890-8567(09)66566-7

[B141] Fombonne E du Mazaubrun C Prevalence of infantile autism in four French regions Soc Psychiatry Psychiatr Epidemiol 1992 27 203 210 10.1007/BF00789007 1411750

[B142] Steinhausen HC Göbel D Breinlinger M Wohlleben BJ A community survey of infantile autism Am Acad Child Psychiatry 1986 25 186 189 10.1016/S0002-7138(09)60225-9 3486202

[B143] Magnússon P Saemundsen E Prevalence of autism in Iceland J Autism Dev Disord 2001 31 153 163 10.1023/A:1010795014548 11450814

[B144] McCarthy P Fitzgerald M Smith MA Prevalence of childhood autism in Ireland Ir Med J 1984 77 129 130 6610668

[B145] Sugiyama T Abe T The prevalence of autism in Nagoya, Japan: a total population study J Autism Dev Disord 1989 19 87 96 10.1007/BF02212720 2708306

[B146] Matsuishi T Shiotsuki Y Yoshimura K Shoji H Imuta F Yamashita F High prevalence of infantile autism in Kurume City, Japan J Child Neurol 1987 2 268 271 10.1177/088307388700200406 3498744

[B147] Tanoue Y Oda S Asano F Kawashima K Epidemiology of infantile autism in southern Ibaraki, Japan: differences in prevalence in birth cohorts J Autism Dev Disord 1988 18 155 166 10.1007/BF02211943 3410807

[B148] Ishii T Takahashi O The epidemiology of autistic children in Toyota, Japan: Prevalence Jpn J Child Adolesc Psychiatry 1983 24 311 321

[B149] Hoshino Y Kumashiro H Yashima Y Tachibana R Watanabe M The epidemiological study of autism in Fukushima-ken Folia Psychiatr Neurol Jpn 1982 36 115 124 7129258 10.1111/j.1440-1819.1982.tb00262.x

[B150] Honda H Shimizu Y Misumi K Niimi M Ohashi Y Cumulative incidence and prevalence of childhood autism in children in Japan Br J Psychiatry 1996 169 228 235 10.1192/bjp.169.2.228 8871801

[B151] Sponheim E Skjeldal O Autism and related disorders: epidemiological findings in a Norwegian study using ICD-10 diagnostic criteria J Autism Dev Disord 1998 28 217 227 10.1023/A:1026017405150 9656133

[B152] Gillberg C Steffenburg S Schaumann H Is autism more common now than ten years ago? Br J Psychiatry 1991 158 403 409 10.1192/bjp.158.3.403 1828000

[B153] Steffenburg S Gillberg C Autism and autistic-like conditions in Swedish rural and urban areas: a population study Br J Psychiatry 1986 149 81 87 10.1192/bjp.149.1.81 3779317

[B154] Gillberg CJ Infantile autism and other childhood psychoses in a Swedish urban region. Epidemiological aspects. Child Psychol Psychiatry 1984 25 35 43 10.1111/j.1469-7610.1984.tb01717.x 6607262

[B155] Bohman M Bohman I Björck P Sjöholm E Schmidt MH, Remschmidt H Childhood psychosis in a Northern Swedish county: some preliminary findings from an epidemiological survey Epidemiological approaches in child psychiatry II 1983 New York: Thieme-Stratton 163 173

[B156] Arvidsson T Danielsson Forsberg Gillberg Autism in 3-6-year-old children in a suburb of Goteborg, Sweden Autism 1997 1 2163 2173

[B157] Kadesjö B Gillberg C Hagberg B Brief report: autism and Asperger syndrome in seven-year-old children: a total population study J Autism Dev Disord 1999 29 327 331 10.1023/A:1022115520317 10478732

[B158] Van Naarden BK Schieve L Daniels J Durkin M Giarelli E Kirby RS Lee LC Newschaffer C Nicholas J Pinto-Martin J Relationships between multiple births and autism spectrum disorders, cerebral palsy, and intellectual disabilities: autism and developmental disabilities monitoring (ADDM) network-2002 surveillance year Autism Res 2008 1 266 274 10.1002/aur.41 19360679

[B159] Yeargin-Allsopp M Rice C Karapurkar T Doernberg N Boyle C Murphy C Prevalence of autism in a US metropolitan area JAMA 2003 289 49 55 10.1001/jama.289.1.49 12503976

[B160] Ritvo ER Freeman BJ Pingree C Mason-Brothers A Jorde L Jenson WR McMahon WM Petersen PB Mo A Ritvo A The UCLA-University of Utah epidemiologic survey of autism: prevalence Am J Psychiatry 1989 146 194 199 2783539 10.1176/ajp.146.2.194

[B161] Burd L Fisher W Kerbeshian J A prevalence study of pervasive developmental disorders in North Dakota J Am Acad Child Adolesc Psychiatry 1987 26 700 703 10.1097/00004583-198709000-00014 3499432

[B162] Treffert DA Epidemiology of infantile autism Arch Gen Psychiatry 1970 22 431 438 10.1001/archpsyc.1970.01740290047006 5436867

[B163] Croen LA Grether JK Selvin S The epidemiology of mental retardation of unknown cause Pediatrics 2001 107 E86 10.1542/peds.107.6.e86 11389284

[B164] Stosic R Dunagan F Palmer H Fowler T Adams I Responsible self-medication: perceived risks and benefits of over-the-counter analgesic use Int J Pharm Prac 2011 4 236 245 10.1111/j.2042-7174.2011.00097.x 21733011

[B165] Werler MM Louik C Mitchell AA Epidemiologic analysis of maternal factors and amniotic band defects Birth Defects Res A Clin Mol Terato 2003 67 68 72 10.1002/bdra.10001 12749386

[B166] Jensen MS Rebordosa C Thulstrup AM Toft G Sørensen HT Bonde JP Henriksen TB Olsen J Maternal use of acetaminophen, ibuprofen, and acetylsalicylic acid during pregnancy and risk of cryptorchidism Epidemiology 2001 21 779 785 20805751 10.1097/EDE.0b013e3181f20bed

[B167] Rebordosa C Kogevinas N Sorensen HT Olsen J Pre-natal exposure to acetaminophen and risk of wheezing and asthma in children: a birth cohort study Am J Respir Crit Care 2008 37 583 590 10.1093/ije/dyn070 18400839

[B168] Andersen AB Farkas DK Mehnert F Ehrenstein V Erichsen R Use of prescription paracetamol during pregnancy and risk of asthma in children: a population-based Danish cohort study Clin Epidemiol 2012 4 33 40 22355259 10.2147/CLEP.S28312 PMC4614522

[B169] Bisgaard H Loland L Holst KK Pipper CB Prenatal determinants of neonatal lung function in high-risk newborns J Allergy Clin Immunol 2009 123 651 657 10.1016/j.jaci.2008.11.036 19152964

[B170] Thulstrup AM Sørensen HT Nielsen GL Andersen L Barrett D Vilstrup H Olsen J Fetal growth and adverse birth outcomes in women receiving prescriptions for acetaminophen during pregnancy. EuroMap Study Group Am J Perinatol 1999 16 321 326 10.1055/s-2007-993879 10614698

[B171] Shaheen SO Newson RB Henderson AJ Headley JE Stratton FD Jones RW Strachan DP ALSPAC Study Team Prenatal acetaminophen exposure and risk of asthma and elevated immunoglobulin E in childhood Clin Exp Allergy 2005 35 18 25 10.1111/j.1365-2222.2005.02151.x 15649261

[B172] Heikkilä AM Erkkola RU Nummi SE Use of medication during pregnancy–a prospective cohort study on use and policy of prescribing Ann Chir Gynaecol Suppl 1994 208 80 83 8092781

[B173] Lee HJ Han JY Yook JH Choi JS Ahn HK Kim MY Song IO Yang JH Nava-Ocampo AA A prospective cohort study of pregnancy outcomes of women inadvertently exposed to methylephedrine in the 1st trimester of pregnancy J Obstet Gynaeco 2010 30 563 566 10.3109/01443615.2010.487577 20701502

[B174] Goksör E Thengilsdottir H Alm B Norvenius G Wennergren G Prenatal paracetamol exposure and risk of wheeze at preschool age Acta Paediatr 2011 100 1567 1571 10.1111/j.1651-2227.2011.02403.x 21767300

[B175] Kang EM Lundsberg LS Illuzzi JL Bracken MB Prenatal exposure to acetaminophen and asthma in children Obstet Gynecol 2009 114 1295 1306 10.1097/AOG.0b013e3181c225c0 19935033 PMC3237391

[B176] Persky V Piorkowski J Hernandez E Chavez N Wagner-Cassanova C Vergara C Pelzel D Enriquez R Gutierrez S Busso A Prenatal exposure to acetaminophen and respiratory symptoms in the first year of life Ann Allergy Asthma Immunol 2008 101 271 278 10.1016/S1081-1206(10)60492-9 18814450 PMC2578844

[B177] Perzanowski MS Miller RL Tang D Ali D Garfinkel RS Chew GL Goldstein IF Perera FP Barr RG Prenatal acetaminophen exposure and risk of wheeze at age 5 years in an urban low-income cohort Thorax 2010 65 118 123 10.1136/thx.2009.121459 19850963 PMC2876309

[B178] Ognjanovic S Blair C Spector LG Robison LL Roesler M Ross JA Analgesic use during pregnancy and risk of infant leukaemia: a Children’s Oncology Group study Br J Cancer 2011 104 532 536 10.1038/sj.bjc.6606046 21157452 PMC3049556

[B179] Cleaves MA Savell VH Jr Raj S Zhao W Correa A Werler MM Hobbs CA National Birth Defects Prevention Study. Maternal use of acetaminophen and nonsteroidal anti-inflammatory drugs (NSAIDs), and muscular ventricular septal defects Birth Defects Res A Clin Mol Teratol 2004 70 107 113 10.1002/bdra.20005 15039924

[B180] Li DK Liu L Odouli R Exposure to non-steroidal anti-inflammatory drugs during pregnancy and risk of miscarriage: population based cohort study BMJ 2003 327 368 10.1136/bmj.327.7411.368 12919986 PMC175811

[B181] Lacroix I Hurault C Sarramon MF Guitard C Berrebi A Grau M Albouy-Cossard C Bourrel R Elefant E Montastruc JL Damase-Michel C Prescription of drugs during pregnancy: a study using EFEMERIS, the new French database Eur J Clin Pharmacol 2009 65 839 846 10.1007/s00228-009-0647-2 19365629

[B182] Lacroix I Damase-Michel C Lapeyre-Mestre M Montastruc JL Prescription of drugs during pregnancy in France Lancet 2000 356 1735 1736 10.1016/S0140-6736(00)03209-8 11095263

[B183] Mikou S Buire AC Trenque T Over the counter medication in pregnant women Therapie 2008 63 415 418 10.2515/therapie/2008064 19236832

[B184] Beyens M-N Prescription of drugs to pregnant women in France: the HIMAGE study Therapie 2003 58 505 10.2515/therapie:2003082 15058494

[B185] Crespin S Bourrel R Hurault-Delarue C Lapeyre-Mestre M Montastruc JL Damase-Michel C Drug prescribing before and during pregnancy in south west France: a retrolective study Drug Saf 2011 34 595 604 10.2165/11589170-000000000-00000 21663335

[B186] Egen-Lappe V Hasford J Drug prescription in pregnancy: analysis of a large statutory sickness fund population Eur J Clin Pharmacol 2004 60 659 666 10.1007/s00228-004-0817-1 15480609

[B187] Cleary BJ Butt H Strawbridge JD Gallagher PJ Fahey T Murphy DJ Medication use in early pregnancy-prevalence and determinants of use in a prospective cohort of women Pharmacoepidemiol Drug Saf 2010 19 408 417 20099251 10.1002/pds.1906

[B188] Bakkeheim E Mowinckel Carlsen Håland Lødrup Carlsen Paracetamol in early infancy: the risk of childhood allergy and asthma Acta Paediatr 2010 100 90 96 21143295 10.1111/j.1651-2227.2010.01942.x

[B189] Nordeng H Ystrøm E Einarson A Perception of risk regarding the use of medications and other exposures during pregnancy Eur J Clin Pharmacol 2010 66 207 214 10.1007/s00228-009-0744-2 19841915

[B190] Ystrom E Vollrath ME Nordeng H Effects of personality on use of medications, alcohol, and cigarettes during pregnancy Eur J Clin Pharmacol 2012 68 845 851 10.1007/s00228-011-1197-y 22189674

[B191] Chen YC Tsai CH Lee Y Gestational medication use, birth conditions, and early postnatal exposures for childhood asthma Clin Dev Immunol 2012 2012 913426 22203862 10.1155/2012/913426 PMC3235498

[B192] Leung KY Lee YP Chan HY Lee CP Tang MH Are herbal medicinal products less teratogenic than Western pharmaceutical products Acta Pharmacol Sin 2002 23 1169 1172 12466056

[B193] Morris BJ Circumcision in Australia: prevalence and effects on sexual health Int J STD AIDS 2007 18 69 70 17326871 10.1258/095646207779949943

[B194] Canadian Circumcision Rate- Mid 1990’s Canadian Institute for Health Information http://www.cihi.ca/CIHI-ext-portal/internet/EN/Home/home/cihi000001

[B195] Morris BJ Waskett JH Gray RH Does sexual function survey in Denmark offer any support for male circumcision having an adverse effect? Int J Epidemiol 2012 41 310 312 10.1093/ije/dyr180 22422464 PMC3383191

[B196] Rickwood AMK Kenny SE Donnell SC Towards evidence based circumcision of English boys: survey of trends in practice Brit Med J 2000 321 792 793 10.1136/bmj.321.7264.792 11009516 PMC27490

[B197] Leung M Tang P Chao N Lui K Hong Kong Chinese parents’ attitude towards circumcision Hong Kong Med J 2012 18 496 501 23223650

[B198] Kim D Koo SA Pang MG Decline in male circumcision in South Korea BMC Public Health 2012 12 1067 10.1186/1471-2458-12-1067 23227923 PMC3526493

[B199] Hsieh TF Chang CH Chang SS Foreskin development before adolescence in 2149 schoolboys Int J Urol 2006 13 9680970 10.1111/j.1442-2042.2006.01449.x 16882064

[B200] National Hospital Discharge Survey, National Center for Health Statistics, Centers for Disease Control and Prevention Estimated number of male newborn infants, and percent circumcised during birth hospitalization, by geographic region: United States, 1979–2008 http://www.cdc.gov/nchs/data/nhds/9circumcision/2007circ9_regionracetrend.pdf

[B201] Wirth JL Current circumcision practices: Canada Pediatrics 1980 66 705 8 7432876

[B202] Frisch M Friis S Kjaer SK Melbye M Falling incidence of penis cancer in an uncircumcised population (Denmark 1943–90) BMJ 1995 311 1471 10.1136/bmj.311.7018.1471 8520335 PMC2543732

[B203] Schoen EJ Kandeel FR, Lue TF, Pryor JL, Swerdloff RS Male circumcision. Male Sexual Dysfunction. Pathophysiology and Treatment 2007 New York: Informa 95 107

[B204] France Male Circumcision rate - Le site sante’ du Minist’ere des Affaires socials et del la Sante’ http://www.sante.gouv.fr/IMG/pdf/er754.pdf

[B205] Kamtsiuris P Bergmann E Rattay P Schlaud M Use of medical services. Results of the German health interview and examination survey for children and adolescents (KiGGS) Bundesgesundheitsblatt Gesundheitsforschung Gesundheitsschutz 2007 50 836 850 10.1007/s00103-007-0247-1 17514470

[B206] Groves H Bailie A McCallion W Childhood Circumcision in Northern Ireland: a barometer of the current practice of general paediatric surgery Ulster Med J 2010 79 80 81 21116424 PMC2993140

